# Oral-Health-Related Quality of Life in Elderly Edentulous Patients with Full-Arch Rehabilitation Treatments: A Systematic Review

**DOI:** 10.3390/jcm13123391

**Published:** 2024-06-10

**Authors:** Tin Thinzar Linn, Angkoon Khaohoen, Khaing Myat Thu, Pimduen Rungsiyakull

**Affiliations:** 1 Department of Prosthodontics, Faculty of Dentistry, Chiang Mai University, 239, Huay Kaew Road, Muang District, Chiang Mai 50200, Thailand; tinthinzarlinn_tin@cmu.ac.th (T.T.L.); angkoon_khaohoen@cmu.ac.th (A.K.); 2 Faculty of Dentistry, The University of Hong Kong, Hong Kong SAR 999077, China; khaing@hku.hk

**Keywords:** elderly, aged, edentulous, full-arch oral rehabilitation, dentures, dental prosthesis, implant supported, oral-health-related quality of life

## Abstract

**Background**: The improvement of oral-health-related quality of life (OHRQoL) with different types of prosthesis for completely edentulous jaws in the elderly population is a critical factor in clinical decision making for these vulnerable patients. This review aims to evaluate the changes in OHRQoL after treatment with different types of full-arch prostheses in the elderly edentulous population to determine the prostheses that result in the greatest improvement in OHRQoL. **Materials and Methods**: Clinical studies of different types of full-arch prostheses that measured the OHRQoL in edentulous patients 60 years or older were searched for in the PubMed, Embase and Scopus electronic databases, with additional hand searching to summarize the outcomes of the selected studies. **Result**: Among the 302 identified studies, 10 studies were selected. A total of 504 patients wearing 133 complete dentures, 372 implant overdentures and 39 fixed prostheses were assessed among the selected studies. The overall OHIP and GOHAI scores were evaluated at baseline and in the 3rd, 6th, 12th and 18th months of treatment with the respective prostheses. The improved OHRQoL with overall OHIP scores associated with conventional dentures were 9.21–12.5% from the 3rd month to 1 year after treatment, whereas those associated with implant overdentures and full-arch fixed prosthesis were 9–25.26% at 1 year and 18.53–26.79 at the 18th-month follow-up, respectively. The increased overall GOHAI scores were 21.3–25.43% for conventional dentures, 36.82–41.32% for implant overdentures and 39.48–42.83% for full-arch fixed prosthesis from the 3rd month to the 6th-month follow-up. **Conclusion**: In general, the improvement in OHRQoL after rehabilitation with implant overdentures declined at one year, and that with full-arch fixed prosthesis declined at the 18th-month follow-up; meanwhile, the OHRQoL associated with conventional dentures improved stably up to one year, but the implant-supported prostheses resulted in an obviously greater improvement in the OHRQoL than that obtained with conventional dentures. However, studies with longer follow-up periods are still required to evaluate the long-term clinical effectiveness.

## 1. Introduction

Treatment decision making for the edentulous elderly is challenging due to their underlying alveolar bone, age-related oral and systemic changes and socioeconomic status. Beyond the physical and physiologic factors, the psychosocial factor is a key factor in considering the appropriate treatment for edentulous jaws because tooth loss surely affects self-esteem. On the other hand, patients’ financial situation usually limits the choice of treatment; therefore, the balance between these factors and improvements in patients’ quality of life should always be considered.

Complete edentulism is a common irreversible disease in aging populations that limits oral functions and psychosocial properties, meaning that their quality of life is negatively affected [1,2]. In the rehabilitation of edentulous jaws, the available options nowadays are a conventional removable complete denture [3] or an implant-supported removable or fixed prosthesis. All types of prostheses rely on the underlying alveolar bones; a complete denture (CD) requires a sufficient alveolar ridge for retention and stability, and an implant prosthesis requires a sufficient ridge volume as well as sufficient bone quality for implant surgery. In elderly patients, prolonged edentulism is common, and this causes the severe resorption of alveolar bone and reduces facial muscular control [4,5]. This condition affects the retention and stability of CDs, especially for individuals with prolonged edentulism and severe alveolar ridge resorption [6,7]. Meanwhile, the implant-supported prosthesis compensates for these limitations with the implant, but the treatment cost is usually higher than that for a CD. Moreover, alternative options such as zygomatic, pterygoid or subperiosteal implants in the posterior maxilla with poor quality and quantity of bone are not always favorable for all patients, especially in edentulous elderly, because extensive surgery, local zygomatic bone abnormalities, chronic sinusitis and soft-tissue- related complications usually limit the treatments.

In the elderly population, age-related oral changes as well as multiple systemic diseases, polypharmacy and a degenerating physiological condition [5,8] sometimes do not favor surgical dental implant treatment; however, this is not a contraindication, with long-term implant survival rates being identified [9,10]. However, the common characteristics of elderly patients, such as declined mobility, dexterity problems, dependency and decreased cognitive abilities, make it difficult for them to attend multiple prolonged and costly dental appointments [11]. Therefore, the factors that need to be considered regarding the choice of prosthesis in the fully edentulous elderly population are not the same as those for older adult edentulous individuals. On the other hand, the key factors for considering the treatment plans, patients’ satisfaction and health-related quality of life differ according to the age of the people receiving the treatments [12,13]. In the elderly, different physical, psychosocial and financial conditions from younger people may influence their choice of full-arch prosthesis, as well as their subjective opinions on the treatments. While the clinical success of different full-arch treatment modalities is evident, the patient-centered parameters associated with these interventions are also increasingly garnering the interest of researchers because their clinical effectiveness cannot be concluded without considering patients’ perception of treatment [14]. Therefore, patient-reported outcome measures such as the patient’s satisfaction and oral-health-related quality of life (OHRQoL) play key roles in the choice of prosthesis for full-arch oral rehabilitation [15]. A recent systematic review comparing patients’ satisfaction and the OHRQoL associated with implant-retained full-arch removable and fixed prostheses showed that patients preferred implant-supported fixed prostheses over implant overdentures in most of the studies [16].

Rehabilitation with a new CD resulted in an improvement in the OHRQoL of the elderly edentulous population [14], but we could not determine the OHRQoL associated with a full-arch fixed prosthesis in the elderly edentulous population by performing a qualitative review. Moreover, recently published qualitative and quantitative reviews of the patient-reported outcomes associated with full-arch implant-supported prostheses do not perform an analysis according to age [16,17]. The age factor is an important determinant of patient-reported outcomes, as patients’ satisfaction and perspective of treatment can vary by age. Moreover, their dependency, cognitive function, willingness and fear of implant treatments may somehow affect patient-reported outcomes. An overview of the subjective outcome measures for full-arch oral rehabilitation treatments would be insightful evidence for the oral care of the elderly population. We also need to provide an overview of studies on all currently available treatment options for full-arch oral rehabilitation in the elderly population, especially those using subjective outcome measures such as OHRQoL. These patient-oriented outcomes have been measured by validated questionnaire-driven assessment methods such as oral health impact profile (OHIP) and geriatric oral health assessment index (GOHAI), which have been widely used as disease-specific measurement tools in geriatric oral health conditions and treatment efficacy [18,19]. Therefore, a qualitative review of OHRQoL associated with all currently available full-arch oral rehabilitation modalities is essential for this homogeneous age group of people (60 years and above). This review aims to evaluate the effects of various full-arch treatment modalities (conventional or implant-supported prostheses, either fixed or removable) on OHRQoL in the elderly edentulous population. It also aims to explore which type of prosthesis is associated with greater improvement of OHRQoL among the three modalities of full-arch rehabilitation.

## 2. Materials and Methods

### 2.1. Study Design 

This systematic review followed the Preferred Reporting System for Systematic Review and Meta-analysis (PRISMA) guidelines and was registered in the International Prospective Register of Systematic Reviews (PROSPERO) (#CRD42023445403) [20]. The review question was “How does the full-arch oral rehabilitation change oral-health-related quality of life after treatment, and which type of full-arch prosthesis results in greater improvement in oral-health-related quality of life in the elderly edentulous population?” The study was structured using the population, intervention, comparison and outcome (PICO) model in which elderly edentulous people (age 60 years and older) as a population received full-arch oral rehabilitation treatments with any type of full-arch prosthesis, either a conventional or implant-supported prosthesis, as an intervention, with pre- and post-treatment periods as a comparison and OHRQoL in which multi-dimensional aspects such as orofacial function, pain and psychological aspect of patients were evaluated subjectively with OHIP and GOHAI as the outcome. 

### 2.2. Eligibility Criteria 

The preset inclusion and exclusion criteria for this systematic review are listed in Table 1.

### 2.3. Literature Search

The search strategy was designed by two accessors (T.L. and A.K.) according to the PICO-based review question. The search terms were set based on medical subject headline (MeSH) terms controlled by a search strategy and combined with the appropriate Boolean operators “OR” or “AND” to perform searches in an electronic search engine. All prospective clinical studies that measured OHRQoL before and after full-arch prosthesis treatments in participants aged 60 years and older were searched for in three electronic databases, namely PubMed, Scopus and Embase, and additional hand searching in the Google search engine until 20 May 2023. The complete set of search terms is displayed in Table 1. 

### 2.4. Study Selection

No restriction was applied in the initial search. Two independent assessors (T.L. and A.K.) performed screening over titles and abstracts for relevancy wherein randomized controlled clinical trials, prospective cohort studies, prospective case–control studies and observational prospective clinical studies in the English language were included. The shortlisted articles were screened with full-text analysis for their eligibility as per preset inclusion and exclusion criteria (Table 1). Disagreements between assessors at each stage were resolved with discussion and the help of a third assessor (P.R.). The entire selection process was conducted using the Internet-based tool Rayyan.

### 2.5. Data Collection

The following data were extracted by two independent accessors (T.L. and A.K.) using a Microsoft Excel spreadsheet containing demographic data of each study, type of jaw being treated (mandibular or maxilla), type of opposing dentition, information about the intervention (conventional denture (CD), implant overdenture (IOD), implant-supported full-arch fixed prosthesis (FD)), type of attachment in IOD, information about implant and mean/medium value of overall OHIP/GOHAI score. Different articles with the same cohort were linked under the same study. Disagreements between assessors were discussed for a consensus, and the third assessor (P.R.) checked the final data to ensure quality.

### 2.6. Risk of Bias Assessment

Risks of methodological bias were assessed by the above-mentioned two assessors independently. The randomized controlled clinical trials (RCTs) were evaluated based on the revised Cochrane risk of bias tool for RCTs (RoB2), and the non-randomized controlled clinical trials (N-RCT) were evaluated with the risk of bias in nonrandomized studies of interventions (ROBINS-I scoring system) [21,22]. Observational prospective studies and studies of pre- vs. post-treatment evaluation without a control group were assessed by the National Institute of Health (NIH) quality assessment tool. 

### 2.7. Data Analysis

Overall mean/medium scores of different OHIP versions and GOHAI of each included study were calculated as percentages to perform a uniform evaluation of all studies. All OHIP versions have 7 domains covering the patient’s perspective of orofacial function, pain, appearance and psychosocial impact, which are usually measured on a four-point Likert scale. Therefore, the % of OHRQoL was calculated in this review using the following formula:

$$\%\;\mathrm{of}\;OHRQoL=\frac{mean\;scores\;of\;OHIPs\;or\;GOHAI}{\left(Total\;number\;of\;respectivequestionnaires\;\times \;4\;or\;6\;points\;likert\;scle\right)}\times 100$$

In our review, the total number of questionnaires was multiplied by 4 for a respective set of OHIP-14, -20, EDENT(19) and 49 assessments to define 100% of OHRQoL. But two studies used a 6-point Likert scale [23,24,25], so the total number of questionnaires used in those 2 studies was multiplied by 6 to define 100%. GOHAI is composed of 12 self-administered questions that cover physical function, psychosocial function, and pain and discomfort function, measured on a 5-point Likert scale which usually ranges from 12 to 60. So, OHRQoL evaluated by GOHAI was calculated as a percentage by multiplying 12 by 5 to define 100% of OHRQoL. The difference in % value between baseline and each timepoint of follow-up for each prosthesis was calculated to evaluate the amount of OHRQoL % change across all studies and between different types of prostheses. Due to the small and low variance of studies and lack of standardization in assessment tools for OHRQoL in included studies, a statistical analysis (meta-analysis) was not conducted. 

## 3. Results

### 3.1. Description of Study and Result of Search

**Figure 1:**
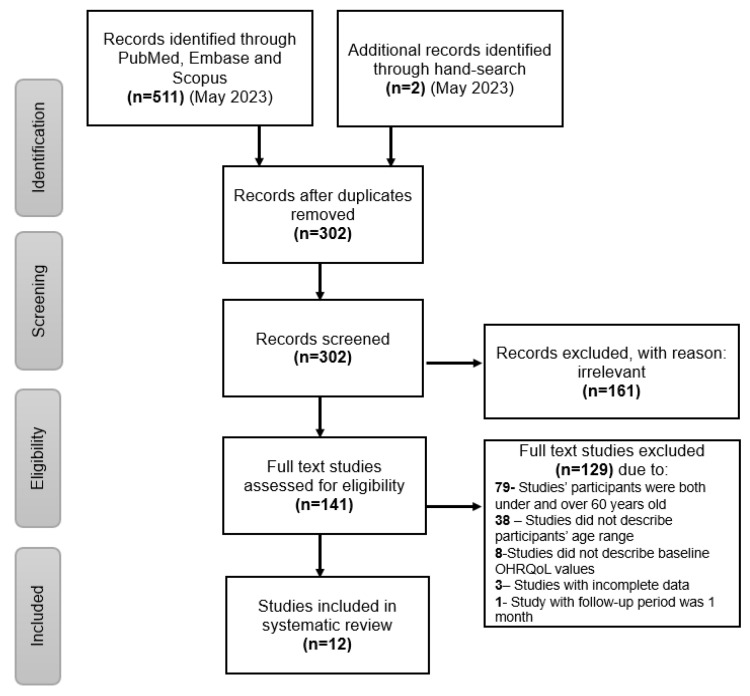
PRISMA flow chart for screening of studies by inclusion and exclusion criteria.

A total of 513 references were retrieved from three electronic databases and through hand searching using PubMed and the Google search engine. After the removal of 211 duplicate articles, the remaining 302 references were screened by title, and the abstracts were read for relevancy. After the removal of 161 irrelevant studies, 141 articles were screened for inclusion with full-text analysis. Consequently, 12 articles were included in this review (Figure 1). 

### 3.2. Methodological Quality of Studies

According to the Cochrane Handbook of Systematic Reviews, version 5.1 [20], after the judgment of the five domains, RCTs that were classified as two out of four have some concerns [26,27], while the other two RCT studies have a low risk of bias (Table 2) [23,24,28]. Two N-RCTs resulted in having a moderate risk of bias due to there being no information about the blinding of the accessor in the measurement of outcome (Table 3) [25,29]. One observational study and three pre- and post-treatment studies without a control group resulted in having fair quality (Table 4) [30,31,32,33].

### 3.3. Study Characteristics and Design

A total of 504 patients aged between 60 and 88 years old were evaluated in this review. All studies were performed across different continents. Five studies were carried out in universities [26,28,29,30,33], one study was performed in a private dental clinic and four studies did not mention their exact place of research [23,24,25,27,31,32]. The maximum follow-up period was up to 18 months. Out of 10 studies, 4 randomized controlled clinical trials [23,24,26,27,28], 2 non-randomized controlled clinical trials [25,29], 1 observational prospective study [30] and 3 pre- and post-treatment studies of different types of full-arch prostheses were involved in this review [31,32,33]. The details of the included studies’ characteristics are described in Table 5.

In total, 133 mandibular CDs opposed by an existing or a new maxillary conventional denture were involved in this study, and 18 dentures were relined to the existing denture. In total, 372 IODs were evaluated in the review. Among the cases, 4 IODs with four retained implants were performed in the maxilla, 178 IODs with one retained implant and 146 IODs with two retained implants were performed in the mandible, and the exact number of retained implants was not specified for the remaining 44 IODs. Most of the overdentures were opposed by complete dentures. A total of 39 FDs were supported by three or five implants, and 6 prostheses were implanted at the maxilla. 

Implant prostheses loaded with delayed loading protocol accounted for 131 prostheses, while immediate loading protocol and conventional loading protocol (6–8 weeks after implant placement) accounted for 96 and 94 prostheses, respectively. Although the loading protocol was not mentioned for 70 prostheses, 20 prostheses were loaded by early loading protocol. The most used retention system for IODs was ball attachment (210 in total), followed by locator (122), magnetic attachment (40) and bar attachment (10).

### 3.4. Oral-Health-Related Quality of Life Associated with Different Treatment Modalities for Fully Edentulous Jaws

When comparing OHRQoL improvement between CDs and implant-supported prostheses, implant-supported prostheses obviously increased OHRQoL after treatment by both OHIP and GOHAI assessment methods. According to the studies evaluated using different OHIP versions, conventional dentures gradually improved OHRQoL up to one year after treatment, after which it started to decline. IODs showed a decrease in OHRQoL at one year, and FDs showed a decrease in OHRQoL at the 18th month. Percentage changes of increase in OHRQoL with OHIP assessments in CDs were 9.21% to 13.52% at the 3rd/4th month, 7.08% at the 6th month and from 7.5% to 12.5% at the 1-year follow-up, as shown in Table 6 [23,24,27,28,31]. For IODs, percentage changes of increase in OHRQoL with OHIP tools were 9% to 26.57% at the 3rd/4th month [25,26,27,28], 12.25% to 41.79% at the 6th month and 18.59% to 25.26% at one year after treatment, as presented in Table 7 [23,24,27,33]. Increases in QHRQoL associated with FDs were reflected in decreases in OHIP values of 18.53%, 32.14% and 26.79% at the 3rd/4th month, 6th month and 18th month, respectively [25,32] (Table 8).

The results of OHRQoL assessed with GOHAI reveal that all types of prostheses resulted in stable improvement of OHRQoL up to 6th months after treatment. Rehabilitation with CDs showed overall OHRQoL values increase of 21.3% at the 3rd month and between 20.67 and 25.43% at the 6th month as presented in Table 6 [29,30]. OHRQoL improvement after rehabilitation with IODs showed a 36.82% increase in overall GOHAI score at the 3rd month and an increase of between 21.38 and 41.32% at the 6th month follow-up period [29,30] (Table 7). For FDs, it was shown that OHRQoL values increased from 39.48% at the 3rd month to 42.83% at 6 months after treatment, as shown in Table 8 [30].

## 4. Discussion

The research hypothesis that all three types of full-arch oral rehabilitation are favorable for the improvement of OHRQoL in elderly patients was supported by this review. In this review, percentage changes of OHRQoL from baseline to respective follow-ups were evaluated and compared between three types of prostheses. Since studies used different assessment formats, the overall evaluation of OHRQoL into a common conclusion was slightly complicated, so an estimated percentage enables all formats of assessment to be calculated into a common platform of evaluation. But the percentage amounts in calculations of different versions of OHIP and GOHAI are very different because of the different numbers of questionaries between OHIP and GOHAI and because scoring in OHIP was by either a 4- or 6-point Likert scale while a 5-point Likert- cale was used in GOHAI.

OHRQoL associated with CDs improved gradually up to 1 year after treatment [23,24,27,28,29,30,31]. IODs also showed obvious improvement of OHRQoL up to a 1-year follow-up with increasing scores. However, in one study, FDs showed a decreased OHRQoL after the 18th-month follow-up [32]. In a comparison among different prostheses, the amount of OHRQoL changes measured with OHIP versions for IODs and FDs is almost double the percentage of those for CDs. Implant-supported prostheses showed greater improvement of OHRQoL than that shown by CDs at each respective follow-up. But comparisons between IODs and FDs show no obvious difference. This finding is in agreement with studies that proved OHRQoL with FDs were not significantly more improved than those with IODs [17]. Another study showed similar OHRQoL and patient satisfaction between IODs and FDs, and one quantitative analysis proved that OHRQoL associated with FDs increased more than that with IODs [16,34].

It is well documented that implant prostheses perform better than conventional dentures in clinical and patient-reported outcomes in edentulous patients [35,36], which might most likely be associated with improved retention and stability of implant prostheses. Since retention and stability of removable prostheses are related to OHRQoL and most of the function and patients’ feelings are dependent on those factors [37], the patient-reported outcomes should be considered as dependent on the underlying bone condition for CDs and IODs, especially in elderly who usually have reduced alveolar bone height [38,39,40].

Meanwhile, the scores of OHRQoL among the general population and elderly have been reviewed. In a systematic review of IOD and FD treatments on patient-reported outcomes in non-specified age groups [41], the mean OHIP-49 scores were 18.9 to 26.5, mean OHIP-14 scores were 1.9 to 3.5 and mean OHIP-EDENT scores were 0.0 to 35.0. In this review, mean OHIP-49 scores were 12.6 to 63.3, mean OHIP-14 scores were 0 to 8.44 and mean OHIP-EDENT scores were 20.9 in several follow-ups. These data show that the upper limit of the mean OHIP scores of elderly groups is greater than that in non-specified age groups, which meant relatively lower OHRQoL in elderly patients with IOD and FD treatments. The reason for this could be construed as a difference in patient expectations and satisfaction as well as the influence of age on OHRQoL, which is directly related to patient satisfaction and preference [42].

Patient preference was found to be directly related to the pain and oral dysfunction domain of OHIP EDENT, indicating that the comfortability of prosthesis is key for treatment outcomes [43]. Some patients preferred FDs because of their high stability, but others preferred removable dentures because of their easiness of cleaning [44]. Additionally, dependency and limited financial capacity make elderly people prefer IODs over FDs in the elderly population [45].

In considering treatment outcomes of prosthodontic treatment, age is also an important factor because patient-reported outcomes and psychosocial outcomes varied with age though clinical outcomes did not. In a study of patient-reported outcomes according to age difference, age affects the psychosocial, global and physical pain domains in GOHAI and OHIP EDENT assessments, revealing that the perception of treatment effect is greater in patients <65 years of age. That study suggested that younger patients with a shorter edentulism period have higher expectations and greater resilience to treatment [46,47,48]. Therefore, this highlights the need for the elderly’s opinion on OHRQoL with edentulous oral rehabilitations.

However, there are several limitations in this review, including inconsistent assessment methods among included studies, and that different versions of OHIP are the most common tools of assessment found in this review. Different scoring systems between OHIP and GOHAI cause limitations in interpreting the results into a common solution. Additionally, the numbers of studies for each type of prosthesis were not balanced, preventing a comparison and conclusion on a clear-cut suggestion for a treatment plan. Moreover, the types of dentitions, which are not uniform across the studies, may have had a particular effect on the overall evaluation. In this review, all studies except one assessed OHRQoL after rehabilitation with CDs for both arches [23,24,28,29,30,31]. While OHIP covers seven different categories such as functional, physical pain, psychological and social discomfort, this review evaluated only the overall OHIP score because not all the included studies provided individual OHIP sub-scores. Nonetheless, an assessment of each subcategory can provide patient impressions of the types of treatment in fully edentulous elderly patients.

Moreover, in this review, OHRQoL associated with removable full-arch treatment should be explored more in correlation with the underlying bone conditions of participants. Additionally, the effect of different attachment types, implant numbers and types of loading protocols should be considered for OHRQoL in IOD treatment. Furthermore, the quality of studies resulted in some concerns and a moderate risk of bias [25,26,27,29]. The methodological qualities of the involved studies ranged from low to moderate risk of bias, meaning that the certainty of evidence ranges from low risk to moderate risk of bias/fair qualities. Furthermore, the most common follow-up period in this review is 6 months; therefore, the long-term changes in OHRQoL in all edentulous therapies should also be investigated.

Despite its limitations, this review offers evidence to support decision making in prosthodontic treatment planning for edentulous elderly patients. A patient’s perspective on full-arch rehabilitation treatment is crucial for choosing the optimal treatment, and it also guides future research directions in the context of an increasingly aging society. Studies with large sample sizes examining the long-term effect of full-arch implant treatments with subgroups of cohorts such as varying numbers of implants and types of attachments on OHRQoL in fully edentulous elderly people should be performed. Furthermore, OHRQoL related to these treatments should be correlated with other parameters such as nutritional deficiency and cognitive function, which are also common problems in fully edentulous elderly people. In addition, OHRQoL associated with full-arch implant prostheses in the maxilla needs to be explored further.

## 5. Conclusions

Within the limitations, the following conclusions are drawn:

▪ CDs improve OHRQoL in general and can stably improve OHRQoL outcomes up to 12 months of follow-up, but less than implant prostheses do.
▪ IODs and FDs improve OHRQoL much more than CDs. IODs showed a decrease in OHRQoL at one year, and FDs showed a decrease in OHRQoL at the 18th month.
▪ The outcome values between IODs and FDs showed no obvious difference in improving OHRQoL.
▪ Long-term assessment is still questionable, and high-quality, long-term follow-up clinical trials are still warranted.

## Figures and Tables

**Table 1:** Inclusion and exclusion criteria, information source, search terms, search strategy.

**Criteria**	Inclusion Criteria	▪ Clinical studies of full-arch prostheses (either conventional denture, implant overdenture or full-arch fixed prosthesis) with or without a comparison group in which participant age was 60 years and older. ▪ Clinical studies of full-arch prostheses that assessed OHRQoL associated with intervention both before and after treatment. ▪ Clinical studies of full-arch prosthesis in which samples had at least 10 participants and a follow-up period of 3 months. ▪ Studies published in English language.
	Exclusion criteria	Studies of full-arch prosthesis in which not all participants are 60 years and older. Studies that mentioned only the mean age as a whole participant group age. Studies that did not mention the baseline value of OHRQoL associated with intervention. Studies in which participants age was 60 years and older but medically compromised. Studies that did not mention the mean/medium overall score of OHRQoL associated with full-arch prosthesis. In vitro studies and finite element studies. Retrospective study, case report, case series, review articles. Studies in other languages.
**Information Sources**	Electronic databases	PubMed, Embase, Scopus
	Journals	Peer-reviewed dental journals available on PubMed, Embase, Scopus databases
	Others	Google
**Search Terms**	Population	#1—(“Aged”[Mesh] OR “Octogenarians”[Mesh] OR “elderly” OR “geriatric” OR “older adults”) #2—(“Jaw, Edentulous”[Mesh] OR “Mouth, Edentulous”[Mesh] OR “fully edentulous” OR “fully edentulous maxilla” OR “fully edentulous mandible” OR “complete edentulous mandible” OR “complete edentulous maxilla”)
	Intervention	#4—(“Dental Implants”[Mesh] OR “Dental Prosthesis, Implant-Supported”[Mesh] OR “Denture, Overlay”[Mesh] OR “implant overdenture” OR “implant supported overdenture” OR “implant assisted overdenture” OR “full arch fixed prosthesis” OR “fixed complete denture” OR “Denture, Complete”[Mesh] OR “removable complete denture”)
	Outcome	#5—(“patient reported outcome measures” OR “PROMs” OR “oral health-related quality of life” OR “OHRQoL” OR “Quality of Life”[Mesh])
**Search Builder**		#1 AND #2 AND #3 AND #4 AND #5
**Search Date**		20 May 2023

**Table 2:** Cochrane risk of bias for randomized controlled trials (RoB2).

	D1	D2	D3	D4	D5	Overall
Heydeck et al., 2003 [23,24]	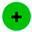	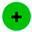	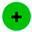	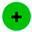	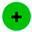	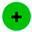
Grovert et al., 2012 [28]	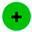	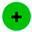	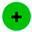	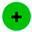	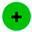	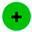
Muller et al., 2013 [27]	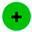	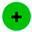	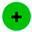	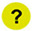	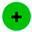	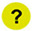
Swindling et al., 2018 [26]	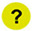	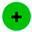	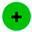	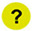	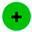	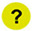

D1—bias due to randomization process; D2—bias due to deviation from indented intervention; D3—bias due to missing outcome data; D4—bias in measurement of outcome; D5—bias in selection of reporting result (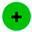, low risk of bias; 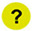, some concern).

**Table 3:** Cochrane risk of bias for non-randomized controlled trials (ROBIN-I).

	D1	D2	D3	D4	D5	D6	D7	Overall
Beresfold et al., 2018 [25]	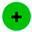	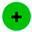	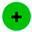	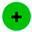	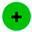	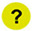	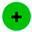	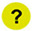
Kiliac et al., 2021 [29]	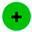	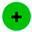	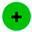	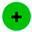	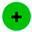	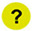	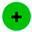	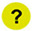

D1—bias in randomization process; D2—bias in selection of participants into the study; D3—bias in classification of intervention; D4—bias due to deviation from intended intervention; D5—bias due to missing data; D6—bias in measurement of outcome; D7—bias in selection of reported results (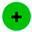, low risk of bias; 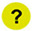, moderate risk of bias).

**Table 4:** Risk of bias assessment for pre–post studies and observational prospective study using respective assessment (Y—yes; N—no; CD—cannot be determined; NR—not reported; NA—not applicable).

Studies	Q1	Q2	Q3	Q4	Q5	Q6	Q7	Q8	Q9	Q10	Q11	Q12	Q13	Q14	Total Y	Quality
NHI quality assessment tools for pre–post studies (12 questions)																
Berretin-felix et al., 2008 [32]	Y	N	Y	NR	CD	Y	Y	NR	NR	Y	Y	NA			7/12	Fair
Geckili et al., 2011 [33]	Y	Y	Y	Y	NR	Y	Y	NR	Y	Y	N	NA			9/12	Fair
Yamamoto et al., 2018 [31]	Y	Y	Y	Y	NA	Y	Y	N	Y	Y	NA	NA			8/12	Fair
NHI quality assessment tools for observational prospective study (14 questions)																
EI Osta et al., 2017 [30]	Y	Y	NR	Y	N	Y	Y	NA	Y	Y	Y	NR	Y	CD	9/14	Fair

**Table 5:** Characteristics of included studies.

Demographics							Jaws and Prostheses			Implant						
Year	Study	Study Design	Follow-Up	Sample Size	Age Range	Mean Age	Comparison/Prostheses	Edentulous Jaw	Opposing Jaw	Type	Number	Length (mm)	Diameter	Brand	Type of Attachment for IOD	Loading Protocol
2018	Beresfold et al. [25]	Non-randomized crossover trial (NRCT)	4 mos	12	60–81	69 ± 6.46	2 IODs vs. 3 implant-supported FDs	Mandible with adequate bone for 11 mm long, 4 mm diameter implant placement			3	10–16	4–4.3	Nobel Biocare	Locator	Delayed
2021	Kilic et al. [29]	Non-randomized controlled study	6 mos	CD: 10, IOD: 44	65–79	68.35 ± 4.1	CD (10) vs. 2 IODs with magnet (10), ball (12), locator (12) and bar attachment (10) groups	Mandible	CD							Not mentioned
2003	Heydecke et al. [23,24]	RCT	6 mos	CD: 30, IOD: 30	65–75	69.4 for CD, 68.9 for IOD	2 IODs vs. CD	Mandible with adequate bone for implants in anterior region	CD	Transmucosal titanium screw implant	2	10–12		ITI	Ball	4 months after implant insertion
2012	Grovert et al. [28]	RCT	3 mons	10	62–77	70	CD vs. CLSIOD vs. SDALSIOD		CD		1	12	3.7	Zimmer Tapered Swiss Plus SPB12	Magnet	Early loading
2013	Müller et al. [27]	RCT	12 mos	Relined: 18, IOD: 16	over 75 yrs		Reline existing denture vs. IOD	Mandible	CD	SLA surface	2	8	4.1 mm	Straumann	Locator, ball	6 to 8 weeks
2017	Schwindling et al. [26]	RCT	4 mos	IML: 81, DL: 77	60–86	70.4 ± 5.9 for IML, 69.1 ± 6.4 for DL	Immediate loading vs. delayed loading of single IOD	Mandible Class II: 81, Class III: 95	CD		1			Promo Plus	Ball	IML, DL
2008	Berretin-Fleix et al. [32]	Pre- vs. post-trial	18 mos	15	60–76	66	Pre- vs. post-treatment with implant-supported fixed prosthesis (acrylic denture)	Mandible	CD	Self-threading	5		3.75–4 mm	3i-NT Osseotite		Immediate loading
2011	Geckili et al. [33]	Pre- vs. post-trial	6 mos	78	65–82		Pre- vs. post-treatment with 2 IODs	Mandible ACP Classes III and IV	CD		2	13	4.5 mm	Astra Tech	Locator	6 weeks after implant insertion
2018	Yamamoto et al. [31]	Pre- vs. post-trial	3 mos	30	68–82	74.7	Pre- vs. post-treatment with CD	Both arches								
2017	EI Osta et al. [30]	Observational prospective study	6 mos	CCD: 35, Full fixed prostheses in both: 6, 2 IODs in mandible with upper denture: 6, 2 IODs in mandible with 4 IODs in maxilla: 4	60–88	69.39 ± 7.164	Full-mouth CD vs. full-mouth implant-supported FDP vs. lower IOD with opposing CD vs. full-mouth IOD	Both arches			IOD; mandible: 2, maxilla: 4				Ball: 10, locator: 2	Not mentioned

**Table 6:** Total OHIP and GOHAI score at baseline and score difference after treatment associated with conventional denture.

Study	Assessment Instrument	Jaw Type	Baseline	3rd/4th Month	6th Month	1st Year
Heydeck et al., 2003 [23]	OHIP-20	Both arches	56.3 (46.92%)		47.8 (39.83%)	47.3 (39.42%)
Muller et al., 2013 [27]	OHIP-Edent	Mandible	32.9 (43.29%)	25.9 (34.08%)		23.4 (30.79%)
Grovert et al., 2012 [28]	OHIP-49	Both arches	41.1 (20.97%)	18.6 * (9.49%)		
El Osta et al., 2017 [30]	GOHAI	Both arches	32.3 (53.85%)	45.1 * (75.15%)	47.6 * (79.28%)	
Yamamoto et al., 2018 [31]	OHIP-49	Both arches	55 (28.06%)	28.5 * (14.54%)		
Kilic et al., 2021 [29]	GOHAI	Both arches	44.6 (74.33%)		57 * (95%)	

* Shows statistically significant improvement of OHRQoL score at respective post-treatment follow-up.

**Table 7:** Total OHIP and GOHAI score at baseline and score difference after treatment associated with implant overdenture.

Study	Assessment Instrument	Type of Jaw	Baseline	3rd/4th Month	6th Month	1st Year
Heydeck et al., 2003 [23]	OHIP-20	Mandible	53.3 (44.42%)		35 * (29.17%)	31 * (25.83%)
Geckili et al., 2011 [33]	OHIP-14	Mandible	31.84 (56.86%)		8.44 * (15.07%)	
Grovert et al., 2012 [28]	OHIP-49	Mandible	41.1 (20.97%)	8.8 (4.49%)		
			41.1 (20.97%)	12.6 (6.43%)		
Muller et al., 2013 [27]	OHIP-EDENT	Mandible	41.1 (54.08%)	20.9 (27.5%)		21.9 (28.82%)
El Osta et al., 2017 [30]	GOHAI	Mandible alone or both arches	32.3 (53.85%)	54.4 (90.67%)	57.1 (95.17%)	
Beresfold et al., 2018 [25]	OHIP-49	Mandible	109.3 (37.38%)	63.3 * (21.53%)		
Schwindling et al., 2018 [26]	OHIP-49	Mandible	Immediate loading, 51.8 (26.43%)	4th month 33.6 * (17.14%)		
			Delayed loading, 50.7 (25.88%)	4th month 27.7 * (14.13%)		
Kiliac et al., 2021 [29]	GOHAI	Mandible	Magnet, 41.6 (69.33%)		54.9 * (91.5%)	
			Locator, 45.3 (75.42%)		58.08 * (96.8%)	
			Ball, 39.6 (66.1%)		54.4 * (90.68%)	
			Bar, 44.6 (74.33%)		59.3 * (98.83%)	

* Shows statistically significant improvement of OHRQoL score at respective post-treatment follow-up.

**Table 8:** Total OHIP and GOHAI score at baseline and score difference after treatment associated with full-arch implant-supported fixed prosthesis.

Study	Instrument	Type of Jaw	Baseline	3rd/4th Month	6th Month	18th Month
Berretin-Fleix et al., 2018 [32]	OHIP-14	Mandible	18 (32.14%)		0 * (0%)	3 * (5.36%)
EI Osta et al., 2017 [30]	GOHAI	Both arches	32.3 (53.85%)	56 (93.33%)	58 (96.67%)	
Beresfold et al., 2018 [25]	OHIP-49	Mandible	109.3 (37.38%)	54.8 * (18.63%)		

* Shows statistically significant improvement of OHRQoL score at respective post-treatment follow-up.

## Data Availability

The datasets used and/or analyzed during the current study are available from the corresponding author on reasonable request.

## Abbreviations

CD	Conventional complete denture
IOD	Implant-retained overdenture
FD	Implant-supported full-arch fixed prosthesis
OHRQoL	Oral-health-related quality of life
OHIP	Oral health impact profile
GOHAI	Geriatric oral health assessment index
mos	Months
IML	Immediate Loading
DL	Delayed loading
CLSIOD	Conventional-length single-implant overdenture
SDALSIOD	Short-dental-arch-length single-implant overdenture
ACP	American College of Prosthodontics
